# The Effect of a Combined Modified Pectoral and Stellate Ganglion Block on Stress and Inflammatory Response in Patients Undergoing Modified Radical Mastectomy

**DOI:** 10.1155/2022/3359130

**Published:** 2022-06-06

**Authors:** Jun Geng, Jing Wang, Yaowen Zhang, Wenxiang Song, Junjia Zhu, Jianqing Chen, Zhen Wu

**Affiliations:** ^1^Department of Anesthesiology, Jiangyin Hospital Affiliated to Southeast University Medical School, Wuxi, 214400 Jiangsu, China; ^2^Department of General Surgery, Jiangyin Hospital Affiliated to Southeast University Medical School, Wuxi, 214400 Jiangsu, China

## Abstract

**Background and Aims:**

Regional anaesthesia reports to attenuate stress and inflammatory responses associated with surgical resection; however, the effectiveness of combined nerve blocks is less often investigated. We evaluated whether a combination of a pectoral nerve block (PNB) and stellate ganglion block (SGB) is more effective than a PNB alone in reducing these responses in women undergoing modified radical mastectomy (MRM).

**Methods:**

This is a prospective randomized controlled trial. Fifty patients with breast cancer were randomly allocated to receive an ultrasound-guided PNB (*n* = 25, PNB only group) or ultrasound-guided PNB combined with SGB (*n* = 25, combined blockade group). The primary outcome was perioperative plasma level of interleukin- (IL-) 6. Secondary outcomes included perioperative plasma levels of cortisol, glucose, IL-8, and tumour necrosis factor- (TNF-) *α*, pain scores, haemodynamic variables, sleep quality, and complications postsurgery.

**Results:**

The combined blockade group exhibited significantly lower IL-6 and TNF-*α* levels 24 h postsurgery. Cortisol levels were significantly lower in the combined blockade group at the end of the surgery. Glucose levels at the time of incision were lower in the combined blockade group. Pain scores up to 12 h postsurgery were significantly lower in the combined blockade group, which also exhibited better perioperative haemodynamic stability. Patients in the combined blockade group reported better sleep quality on the night of surgery.

**Conclusion:**

In patients undergoing MRM, PNB combined with SGB block effectively blunted perioperative inflammatory response than PNB alone. A combined block approach can also alleviate stress response and postoperative acute pain with stable perioperative haemodynamics and better postoperative sleep quality.

## 1. Introduction

The association between cancer, stress, and inflammatory responses is well-established [1]. Surgical stimulation and general anaesthesia are key variables that affect the surgical stress response and exacerbate perioperative inflammation [2]. Stress response is harmful for postoperative recovery and long-term prognosis of cancer patients [3]. A wide range of inflammatory factors, such as interleukin- (IL-) 6 [4], IL-8 [5], and tumour necrosis- (TNF-) *α* [6], have been shown to promote tumour progression and metastasis.

Pectoral nerve block (PNB) is an alternative to both paravertebral and epidural blockade for anaesthesia during breast surgery and postoperative pain management [7]. Stellate ganglion blockades (SGBs), involving the injection of local anaesthetic around the cervical sympathetic trunk of the stellate ganglion, can be used to facilitate diagnosis and prognosis [8]. SGB can also be used therapeutically in various medical conditions [8]. A previous study reported that a PNB can effectively suppress postoperative pain and stress response in patients who underwent a modified radical mastectomy [7]. While the individual effects of PNB and SGB have been widely investigated, there is lack of evidence pertaining to the effectiveness of their combination in reducing perioperative stress and inflammatory responses [7, 8].

The objective of this study was to evaluate whether the combination of an ultrasound-guided SGB and PNB is more effective than PNB alone in reducing stress and inflammatory responses in women undergoing modified radical mastectomy (MRM).

## 2. Methods

This trial was approved by the Ethics Committee of our hospital (approval number 2018-021) and followed the principles of the Declaration of Helsinki. This study was conducted at a single hospital. Recruitment for this trial occurred between 03 March 2020 and 31 August 2020. The study was registered prior to patient enrolment (Identifier No: ChiCTR2000030285, date of first registration 27/02/2020). All patients provided written informed consent before surgery.

Women with an American Society of Anesthesiologists physical status grade of I–II, aged >18 years, who were scheduled to undergo a modified radical mastectomy with regional block combined with general anaesthesia were eligible for inclusion. Patients were excluded if they refused to participate or had one or more of the following conditions: infection at the injection site, liver and kidney dysfunction, coagulation dysfunction, arrhythmia, mental disorder, thyroid dysfunction, severe cardiopulmonary dysfunction, autonomic or central nervous system dysfunction, history of couplant allergy, distant metastasis of cancer cells, and immune diseases. Additionally, patients who had received radiotherapy and chemotherapy, were on long-term oral sedatives, had preoperative pain, and needed preoperative analgesics were excluded.

A preanaesthetic interview was conducted by a researcher who was blind to group assignment to evaluate the patients' eligibility before randomisation and to record baseline data. A computerised random-number generator was used for randomisation, and patients were randomly allocated to either the combined blockade group or PNB only group at a ratio of 1 : 1. The details of the allocated treatments were contained in serially numbered sealed opaque envelopes. Another researcher (not involved in this trial and not blind to group assignment) opened the envelopes to view the patients' group assignment in the surgical theatre, before block administration and induction of anaesthesia. A third researcher, who was blind to the group allocation, verified the collection of intraoperative data and blood samples. Postoperative data and blood samples were collected by the same researcher who visited the patients before surgery and at 4, 8, 12, 24, 48, and 72 h postsurgery. All surgeries were performed by the same team of surgeons.

Upon arrival in the operating room, patients underwent standard monitoring (i.e., noninvasive arterial blood pressure, electrocardiogram, pulse oximeter, and capnography). Patients in the combined blockade group received an ultrasound-guided SGB with 5 mL of 0.15% ropivacaine [9] and an ultrasound-guided PNB with 20 mL of 0.375% ropivacaine before general anaesthesia. Patients in the PNB only group were only administered an ultrasound-guided PNB with 20 mL of 0.375% ropivacaine before general anaesthesia; no sham intervention (for SGB) was provided.

Both SGB and PNB were conducted by the same anaesthesiologist before general anaesthesia. The ultrasound-guided right-sided SGB with a linear probe was performed with the patient in a supine position, with a thin pillow under the neck. Under ultrasound guidance, the C7 level was determined. Since there was only one tubercle in the transverse process of C7, the probe was slowly moved upward after determining the C7 level. The C6 level corresponded to the point at which the first transverse process with anterior and posterior tubercles was detected. Using a 23-gauge needle, 5 mL of 0.15% ropivacaine (combined blockade group) was injected below the fascia of the longus colli muscle at the C6 level. The appearance of Horner's syndrome (miosis, ptosis, enophthalmos, sweating, and flushing, and the presence of more than three of the above-mentioned signs was considered positive) indicated a successful block.

PNBs were performed on the same side as the surgery by the same anaesthesiologist who performed SGB. The patients were placed in the supine position with their arm abducted at approximately 90°. The needle was inserted, from the lateral to the medial direction, into the plane between the serratus anterior and pectoralis minor in the proximity of the third rib; 20 mL of 0.375% ropivacaine was injected.

General anaesthesia was induced in the same manner for both groups: administration of 0.05 mg/kg midazolam, 0.5 *μ*g/kg sufentanil, 1–2 mg/kg propofol, and 0.15 mg/kg cisatracurium; this was followed by maintenance with cisatracurium, propofol, and sufentanil. Anaesthesia was monitored with the bispectral index- (BIS-) Vista monitor, with the BIS being maintained between 40 and 60 during surgery. An appropriate dose (1–5 *μ*g) of sufentanil was used for intervention when haemodynamic values (mean arterial pressure (MAP) or heart rate (HR)) increased by >15% from baseline (preoperative blood pressure when the patients were calm). A 2 mL blood sample was withdrawn from a peripheral vein and collected in a plasma tube at T0 (upon arrival of the patient in the operating theatre), T1 (immediately postincision), and T2 (at the end of the surgery) for the determination of stress hormone (cortisol) and glucose levels. Another 2 mL blood sample was drawn at T0 (upon arrival of the patient in the operating theatre), T3 (24 h postsurgery), and T4 (72 h postsurgery) to determine the levels of inflammatory factors (IL-6, IL-8, and TNF-*α*). The blood samples were sent to the laboratory on the same day, and the results of the sample analysis were submitted to the first author.

The primary outcome was the plasma levels of IL-6 at different perioperative time points. Secondary outcomes included perioperative stress hormones and inflammatory factors (cortisol, glucose, IL-8, and TNF-*α*), haemodynamic variables during surgery (HR and MAP), pain scores (visual analogue scale (VAS)), sleep quality, and complications during the first two postoperative days. HR was continuously measured, and MAP was measured at the following time points: before anaesthesia, before incision, immediately postincision, 1 h postincision, before the end of the surgery, before tracheal extubation, and 5 min after tracheal extubation. After the surgery, the patients reported their pain intensity on a VAS (0–10, where 0 indicates no pain and 10 is the maximum pain imaginable). VAS scores were assessed and recorded at 4, 8, 12, 24, and 48 h postsurgery in the ward. For patients requiring postoperative analgesics, the name and dosage of the analgesics were recorded; however, the VAS scores after the use of analgesics were not included in the statistical analyses. We used the Athens Insomnia Scale to assess the perioperative sleep quality of the patients [10]. The questionnaires were completed upon arrival at the operation room and on the first day postsurgery to record the sleep quality on the nights before and after surgery, respectively. We also recorded the incidence of relevant short-term complications, including anorexia, nausea, vomiting, dizziness, and edema of the affected upper limb during the hospital stay.

Statistical analyses were performed with SPSS 18.0. Sample size calculation was based on pretest data which was obtained from 10 patients. Pretest results are as follows: the average (mean ± SD) plasma level of IL-6 on postoperative 24 h was 8.813 ± 1.204 pg/mL in the combined blockade group and 10.088 ± 1.512 pg/mL in the PNB only group. Sample size formula is *n*_1_ = *n*_2_ = (2(*z*_*α*/2_ + *z*_*β*_)^2^*σ*^2^)/*ϵ*^2^. Based on sample size analysis performed using PASS 11.0, with a power of 80%, an alpha of 0.05, and a group difference at a level of 0.05 of significance in the IL-6 level, a minimum sample size of 23 participants in each arm was required. Considering approximately a 10% dropout rate, we decided to recruit 25 patients for each group. Normal distribution of the data was analysed with the Shapiro–Wilk test. Data were expressed as means ± standard deviation (SD). Comparisons of cortisol, glucose, IL-6, IL-8, TNF-*α*, VAS pain scores, sleep quality scores, and haemodynamic variables between groups were performed with the repeated-measures analysis of variance. Baseline characteristics were compared between groups with Student's *t*-test, followed by a two-tailed Dunnett test. The level of statistical significance was set at *P* value < 0.05.

## 3. Results

**Figure 1:**
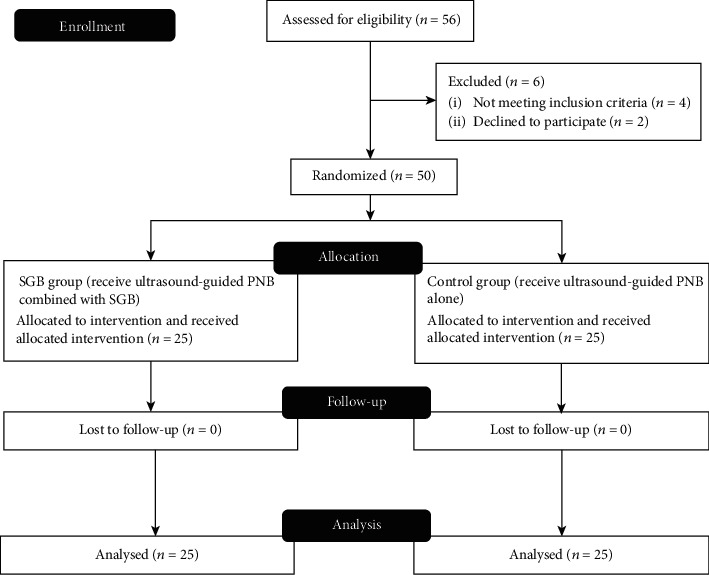
Consort e-flowchart.

A total of 50 patients (25 in each group) were recruited (Figure 1). There were no significant differences in baseline characteristics between the combined blockade group and PNB only group (Table 1). Similarly, the doses of analgesics used during surgery were not significantly different between the two groups.

Plasma levels of IL-6 and TNF-*α* were not significantly different at baseline (T0) but were significantly lower in the combined blockade group at 24 h postsurgery (T3). IL-6 levels were lower in the combined blockade group than in the PNB only group at T3 (Table 2). The IL-6 levels at T3 were significantly higher than those at T0 in both groups (*P* = 1.740 × 10^−5^ in the combined blockade group and *P* = 2.134 × 10^−10^ in PNB only group). TNF-*α* levels were lower in the combined blockade group than in the PNB only group at T3 (Table 2). No significant differences in IL-8 levels were found between the two groups at any time point (Table 2). IL-8 levels at T3 were significantly lower than those at T0 in both groups (*P* = 4.004 × 10^−7^ in the combined blockade group and *P* = 1.049 × 10^−7^ in the PNB only group).

**Figure 2:**
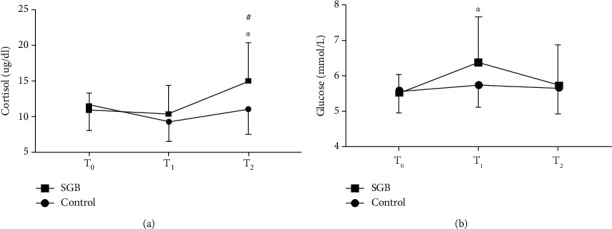
Plasma concentration of cortisol (a) and glucose (b). SGB: stellate ganglion block; T0: baseline; T1: immediately after incision; T2: at the end of surgery. Data are expressed as mean ± SD. ^∗^*P* < 0.05 compared with the PNB only group; ^#^*P* < 0.05 compared with baseline.

Cortisol and glucose levels were not significantly different between the two groups at baseline (T0). At the end of the surgery (T2), cortisol levels were significantly lower in the combined blockade than in the PNB only group (*P* = 0.004) (Figure 2(a)). Compared with baseline, plasma levels of cortisol in the PNB only group were significantly higher at T2 (*P* = 0.001) (Figure 2(a)). Glucose levels were higher in the PNB only group than in the combined blockade group at T1 (*P* = 0.036), while no differences were observed at T0 (Figure 2(b)). In addition, the fluctuation of intraoperative plasma level of glucose was more stable in the combined blockade group than in the PNB only group.

**Figure 3:**
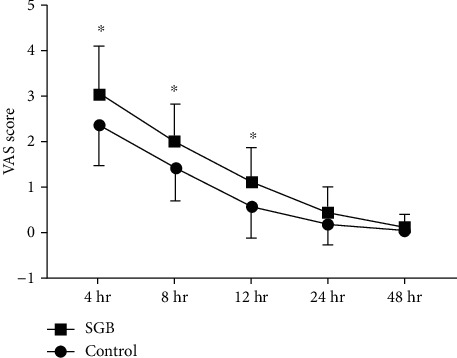
VAS pain scores at the different time points. Time interval is defined as time between baseline and postoperative time. VAS: visual analog scale; SGB: stellate ganglion block. Data are expressed as mean ± SD. ^∗^*P* < 0.05 compared with the PNB only group.

**Figure 4:**
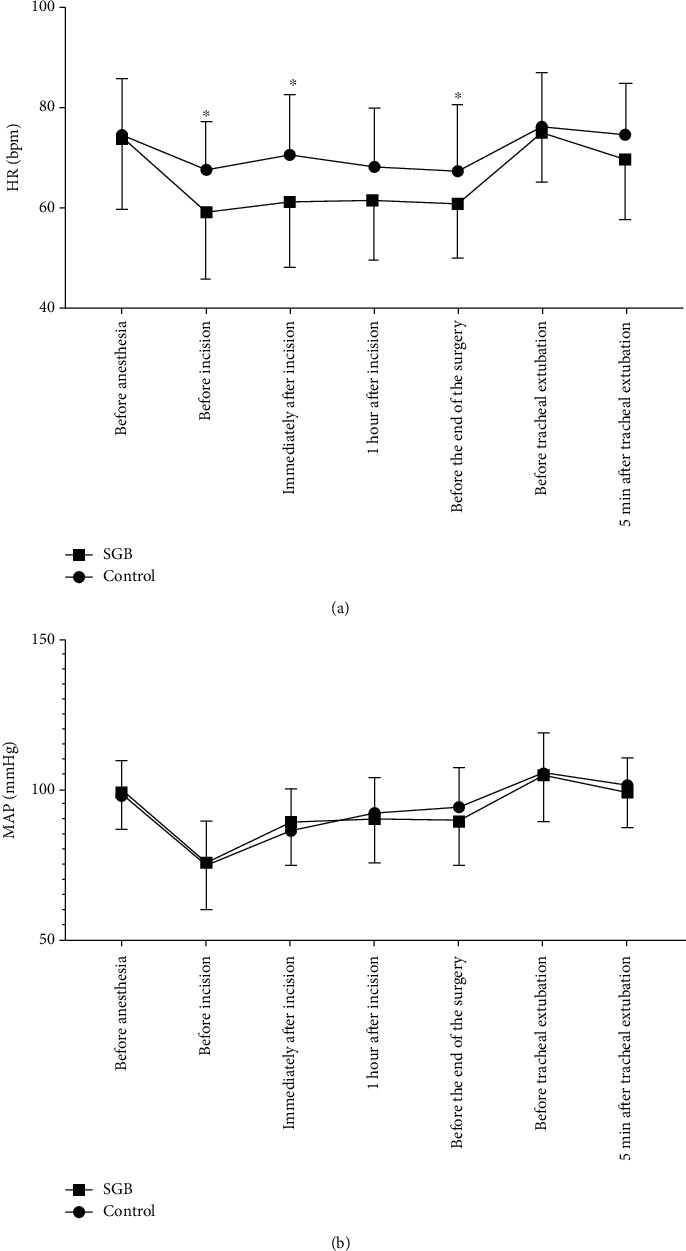
Hemodynamic changes perioperatively. (a) Heart rate (HR). (b) Mean arterial blood pressure (MAP). SGB: stellate ganglion block. Data are expressed as means ± SDs. ^∗^*P* < 0.05 compared with PNB only group.

No postoperative analgesics were required for any of the patients. VAS pain scores at the different time points are shown in Figure 3. Patients in the PNB only group had higher VAS pain scores compared to those in the combined blockade group at 4, 8, and 12 h postsurgery (*P* = 0.019, *P* = 0.008, and *P* = 0.016, respectively); no significant differences were observed at 24 or 48 h postsurgery. Haemodynamic data are shown in Figure 4. Perioperative HR changes were lower in the combined blockade group than in the PNB only group (Figure 4(a)). There were no significant differences in MAP between groups at any time point (Figure 4(b)). Sleep quality scores on the night before surgery were not significantly different between groups; however, scores on the night postsurgery were significantly higher in the PNB only group than in the combined blockade group (*P* = 0.002). The incidence of complications during the first 2 postoperative days is shown in Table 3, and there were no significant differences between the two groups.

## 4. Discussion

The present study reports that the combined block method causes significantly lower postoperative IL-6 and TNF-*α* levels than a PNB alone in patients undergoing MRM. Plasma levels of cortisol and glucose during surgery were also blunted with the combined SGB and PNB approach with stable haemodynamics and no serious side effects.

IL-6 is highly expressed during inflammatory responses to stress and can be used to assess the severity of surgical trauma [11]. Reduction in IL-6 levels in the combined blockade group suggests that the combined block approach can attenuate the inflammatory response. This result is supported by findings from a study conducted by Zhu et al. who reported the ability of the SGB to reduce inflammatory response in patients undergoing laparoscopic colorectal cancer surgery [12]. TNF-*α* is one of the most important mediators of systemic inflammation and is released within a few minutes after local or systemic tissue injury [13]. Therefore, it can be used as an indicator of an early inflammatory reaction [13]. In our trial, the plasma levels of TNF-*α* were significantly lower in the combined blockade group 24 h postsurgery; this may reflect the alleviation of the inflammatory response by the combination of SGB and PNB. IL-8 plays a key role in acute inflammation [14]. While there was no significant difference in IL-8 values between the combined blockade and PNB only groups, we observed a significant reduction in IL-8 from T0 to T1 in both groups. We speculate that this reduction may have been related to preoperative anxiety; however, previous studies have suggested that anxiety may not be significantly associated with inflammatory markers such as IL-8 [15, 16]. Therefore, further research is needed to elucidate the relationship between preoperative anxiety and changes in inflammatory markers.

The neuroendocrine mediators and cytokines through direct activation of the somatic and sympathetic nervous systems can accurately reflect perioperative stress response. Plasma levels of cortisol and glucose are sensitive indicators of the stress response and accurately reflect the stimulus intensity [17, 18]. In our study, cortisol levels were significantly lower at the end of the surgery, while glucose levels were significantly lower immediately postincision in the combined blockade group than in the PNB only group. Our findings are consistent with those of Chen et al. who reported that SGB reduced stress responses in patients undergoing elective laparoscopic cholecystectomy [19].

Previous clinical trials have reported that the SGB can provide effective analgesia [20, 21]. The results of present study confirmed the effectiveness of the SGB for effective analgesia up to 12 hours. As postoperative analgesia reduces the stress response, the analgesic effect of the combined SGB and PNB may partly explain the reduction of cortisol, glucose, and inflammatory factor levels in the present study. Patients in the combined blockade group also exhibited significantly more stable HR changes than those in the PNB only group; similar results were also reported by Chen et al. [19]. In the present study, postoperative sleep quality was significantly higher in the combined blockade group; this supports the use of the combined block approach in patients with perioperative sleep disorders.

There are a few limitations of the present study. Firstly, the sample size was small. Secondly, the use of sufentanil for analgesia during surgery may have influenced the neuroimmuno endocrine network. Furthermore, we did not assess long-term clinical effects of the combined block approach. Therefore, further studies are required to evaluate long-term outcomes such as patient prognosis, cancer migration, and interference with the immune system after the combined use of the SGB and PNB.

## 5. Conclusion

In patients undergoing MRM, PNB combined with SGB block effectively blunted perioperative inflammatory response than PNB alone. A combined block approach can also alleviate stress response and postoperative acute pain with stable perioperative haemodynamics and better postoperative sleep quality.

## Figures and Tables

**Table 1:** Baseline characteristics.

Variable	Combined blockade group (*n* = 25)	PNB only group (*n* = 25)	*P*
Age (years)	54.52 ± 7.81	55.76 ± 9.59	0.620
ASA grade I/II, *n* (%)	4 (16%)/21 (84%)	3 (12%)/22 (88%)	0.691
Height (cm)	158.40 ± 4.86	158.56 ± 4.62	0.907
Weight (kg)	61.78 ± 9.45	58.35 ± 8.22	0.186
Time of operation (min)	102.08 ± 27.75	99.52 ± 19.90	0.715

Data is represented as mean ± SD. ASA: American society of Anesthesiologist physical status.

**Table 2:** Plasma concentration of IL-6, IL-8, and TNF-*α*.

Variable	Time point	Combined blockade group (*n* = 25)	PNB only group (*n* = 25)	*P*
IL-6 (pg/mL)	T0	2.396 ± 0.979	2.656 ± 1.619	0.504
	T3	7.964 ± 4.656	10.876 ± 3.869	0.023^∗^
	T4	3.668 ± 1.610	3.376 ± 1.567	0.527

IL-8 (pg/mL)	T0	105.636 ± 57.102	100.016 ± 48.635	0.715
	T3	34.136 ± 17.439	30.68 ± 24.308	0.574
	T4	31.244 ± 20.452	33.852 ± 26.572	0.705

TNF-*α* (pg/mL)	T0	7 ± 1.381	7.048 ± 2.313	0.931
	T3	8.18 ± 2.144	11.256 ± 3.970	0.002^∗^
	T4	7.296 ± 3.055	7.772 ± 2.140	0.535

^∗^ *P* < 0.05 statistically significant. T0: baseline; T3: 24 hours after the surgery; T4: 72 hours after the surgery. Data are expressed as means ± SD.

**Table 3:** Incidence of complications during the first two postoperative days.

Complications	Combined blockade group (*n* = 25)	PNB only group (*n* = 25)	*P*
Anorexia	0	1	1.000
Nausea	6	6	1.000
Vomit	4	5	1.000
Dizzy	4	9	0.107
Edema of affected upper limb	1	4	0.349

## Data Availability

Dr. Zhen Wu can be contacted to request the data. The e-mail address is drwuzhen2013@163.com.

## Supplementary Materials

Supplementary Materials CONSORT 2010 checklist of information to include when reporting a randomised trial∗.
